# Delivering Trauma-Informed Care in a Hospital Ward for Older Adults With Dementia: An Illustrative Case Series

**DOI:** 10.3389/fresc.2022.934099

**Published:** 2022-07-12

**Authors:** Leah Couzner, Natalie Spence, Karina Fausto, Yan Huo, Lynn Vale, Samantha Elkins, Johanna Saltis, Monica Cations

**Affiliations:** ^1^College of Education, Psychology and Social Work, Flinders University, Adelaide, SA, Australia; ^2^Southern Adelaide Local Health Network, Adelaide, SA, Australia; ^3^Central Adelaide Local Health Network, Adelaide, SA, Australia

**Keywords:** dementia, hospitals, inpatient care, behavior, psychological trauma, mental health, trauma-informed care

## Abstract

**Introduction:**

Up to 70% of older adults have experienced a psychologically traumatic event in their life. Traumatic events can have lifelong effects on functioning and emotion regulation and can affect behavior and experiences in care settings. Common healthcare practices and environments can be re-traumatizing for trauma survivors. These features may trigger behavior change (e.g., aggression and agitation) particularly after the onset of dementia. However, very little research exists to understand how the effects of traumatic events manifest in aged care settings. Trauma-informed care is a framework in which the potential impact of trauma is acknowledged, and practices and procedures are adapted to maximize feelings of control and safety for the patient. Trauma-informed care is an innovative approach with little published evidence in acute geriatric settings.

**Methods:**

We present a series of cases to demonstrate how psychological trauma can affect the experience of inpatient care for older people. The cases detail the patients' relevant background, triggers and behaviors followed by the steps taken by staff to support the patient and respond to their trauma-related needs. These cases describe how the principles of trauma-informed care can be applied to recognize when past psychologically traumatic events are impacting the older adult in hospital. The outcomes of these interventions are reported on in terms of their impact on challenging behavior, patient experiences and satisfaction with care, and/or staff confidence and skill.

**Findings:**

A range of past events negatively impacted the patients during their time in hospital, including childhood abuse, military service, and domestic violence. Staff implemented strategies to accommodate trauma-related needs while providing care that improved safety and reduced patient distress. Principles of trauma-informed care were applied where able, including providing choices and enabling autonomy. However, organizational and environmental features of inpatient wards continued to pose risks for re-traumatisation.

**Conclusions:**

Trauma-informed care is an under-utilized yet potentially beneficial approach to care for older adults in the hospital setting. The cases detailed here demonstrated that the impact of psychological trauma requires an individualized response from staff which when effectively implemented can promote staff and patient safety, reduce the risk of re-traumatisation, and minimize adverse events.

## Introduction

Older people, particularly people with dementia, are frequent users of hospital services. In Australia, the incidence of hospitalization rises with age and peaks at 1,300–1,850 admissions per 1,000 population at age 85 years and older (1). Similar trends are reported internationally (2). Geriatric evaluation and management units were designed to provide comprehensive geriatric assessment and care to older people with and without dementia who have experienced an acute event (e.g., a fall), with multidisciplinary input to minimize disability and reduce the risk of placement in residential aged care (3). In some settings these wards also admit older people with dementia who cannot be cared for at home or in a residential aged care facility because of behavior change that poses risk to the person themselves or people caring for them. The wards provide longer periods of rehabilitation and restorative care and so are distinct from other hospital wards offering short term acute health care (3).

Geriatric inpatient services like geriatric evaluation and management units often retain a biomedical focus with the overall goal to promote return to home as quickly as possible (4). The extent to which mental health needs are assessed and managed varies between settings and patients, but mental health concerns are not the primary focus of treatment in non-psychiatric wards (5).

However, at least 70% of older adults have experienced a psychologically traumatic event in their life (6). While most people who experience psychological trauma recover, traumatic events can have lifelong effects on functioning and emotion regulation and affect behavior and experiences in care settings (7). Older trauma survivors can experience re-traumatisation in care settings that provide reminders of traumatic events and/or present limitations to autonomy, choice, and control that are often essential to recovery (7). In addition, the onset of dementia can trigger the re-emergence of traumatic stress symptoms even where these have been dormant for many years (8, 9). Common hospital care practices (e.g., assisting with toileting and bathing) and environments (e.g., locked wards) have been identified as potential sources of distress for older trauma survivors (10, 11).

Triggering and re-traumatisation can present in the form of aggression, agitation, withdrawal, and other behavior change. These behaviors are usually an external expression of internal fear and distress, but can appear unrelated or unprovoked to care providers (12). Behavioral responses are common in mental health settings where most care recipients have a history of trauma exposure (13). However, the link between traumatic stress and behavioral responses in geriatric and dementia care settings is less well understood. In these settings, behavioral responses commonly result in the use of chemical restraint despite the low efficacy of these medications and their associated side effects (14, 15). Some emerging evidence demonstrates that symptoms of co-morbid post-traumatic stress disorder can be confused for behavioral and psychological symptoms of dementia in geriatric care settings (12).

Trauma-informed care is a framework that originated within mental health services. In trauma-informed care settings, all care professionals are aware of the potential impact of trauma, can identify when a person may be experiencing triggering or re-traumatisation, can adapt practices and procedures to promote autonomy and safety, and provide an environment which maximizes a sense of control and choice for the person (16). Trauma-informed care is widely used in the mental health sector; however, it remains a relatively uncharacterised and innovative approach with little published evidence in geriatric settings (including inpatient settings) (7). The six core principles of trauma-informed care include maximizing the availability of: safety, trustworthiness, choice, collaboration, empowerment, and respect for diversity and inclusion.

In this paper we present a case series of older adults admitted to an inpatient non-psychiatric geriatric ward in 2021 in which historical psychological trauma contributed to symptom presentation. We will illustrate how the application of the principles of trauma-informed models of care by staff in geriatric inpatient care facilitated improved identification and response to trauma-related needs, and associated outcomes in terms of patient wellbeing, experiences of care, and behavior.

## Methods

### Setting

The cases presented here were drawn from three inpatient non-psychiatric geriatric management wards across three hospitals. Two of these wards (51 beds total) include rooms shared by two to four patients and are staffed by medicine, nursing, physiotherapy, occupational therapy, speech pathology, dietetics, and social work professionals. Each ward has a gym area for physical rehabilitation therapy. The third ward is a 12-bed specialized unit for people with dementia and associated behavior change, with single patient rooms and medical, nursing and allied health professional staffing. The staff were engaged in a training and mentoring program to improve the delivery of trauma-informed care within the service (17). However, none of the wards included staffing from psychiatry, psychology, or mental health nursing although consultation could be sought from external mental health services where needed.

### Case Selection

Cases were selected where they illustrated how past psychologically traumatic events may be contributing to the symptom presentation of a person with dementia in an inpatient hospital setting. They were also chosen as they demonstrate a variety of methods by which the principles of trauma-informed care can be successfully applied in this patient population. These cases emerged during usual care upon discussion between clinicians working within the service (KF, NS, YH, SE, and LV) and researchers (MC, LC).

### Analysis and Reporting

The cases are presented in accordance with the CAse REport (CARE) reporting guidelines for case reports in medical journals (18). The guidelines consist of a 13-item checklist of information deemed essential to ensure thoroughness and transparency when writing case reports for publication. The items include, among others, patient information, clinical findings, timeline, therapeutic intervention, and outcomes.

### Ethics

Ethical and governance approval for this study were provided by the Southern Adelaide Clinical Human Research Ethics Committee on 15 May 2020 (REF: HREC/20/SAC/52 and SSA/20/SAC/53). Informed consent was obtained from the next of kin for three of the cases The remaining case had since died and their next of kin was unable to be contacted. The patients featured in the cases presented have been given pseudonyms to protect their anonymity and any identifying information has been omitted. The case for which informed consent was unable to be obtained has been heavily deidentified and anonymised (Case 1).

## Results

### Case 1

Mrs G, a 94-year-old woman, was admitted after a fall at home. Mrs G's medical history included Alzheimer's disease. Her initial admission was several months long in duration, upon which Mrs G was discharged to a residential aged care facility. Mrs G was readmitted to the ward 8 days later due to behaviors that staff at the residential aged care facility found difficult to manage. During both of her admissions, Mrs G would become distressed particularly during personal care. This distress was characterized by verbal and physical aggression including, biting, spitting, punching, and grabbing staff. Four staff were required to assist Mrs G with personal care. Staff reported being afraid of her and would attend to her personal care very quickly. Mrs G's family were distressed by her behavior, telling staff that these reactions were out of character for her. Staff could not identify the triggers for this behavior until a family member asked for a private meeting and disclosed that Mrs G had experienced sexual abuse in the past. Together, family and staff identified that personal care delivered by male staff was triggering for Mrs G, and staff efforts to persist with care were interpreted by Mrs G as disrespectful and untrustworthy.

Staff developed a behavior support plan for Mrs G that included reference to a “traumatic event” in her past, without specific details. The support plan involved reducing exposure to triggers by ensuring only female nursing staff provided personal care to Mrs G, and actions to promote Mrs G's sense of control (e.g., choices in how often and how to wash) and trust in the staff (e.g., transparency in all actions, with clear routines around personal care, and the ability to say “no”). Multiple information sharing systems (e.g., handover, notes, signs) were used to ensure all staff were informed of the strategies included in the plan and the plan was regularly updated as strategies that promoted safety and trust for Mrs G were identified. Upon implementing this approach, Mrs G's distress during personal care settled. After just over a month in the ward, Mrs G was discharged and has not been re-admitted to the ward since. The case triggered a change in practice in which family members were more routinely asked, where appropriate, at ward admission whether any stressful life events may impact care.

### Case 2

Mr H, a 73-year-old male, was born in Holland in 1946. His father died in World War II before his birth, and mother died at childbirth. Mr H was subsequently raised by foster parents with limited social contact throughout childhood. Mr H had a diagnosis of Alzheimer's disease but no other major medical history. He was repeatedly admitted to hospital throughout 2021 because of self-harm behavior in the residential aged care facility in which he lived that was difficult for the staff to manage. Behavior both in hospital and in the residential aged care facility was characterized by anxiety, high levels of dependence on staff, significant distress at signs of abandonment (e.g., care staff attending to another patient) presenting as head banging, scratching, and screaming, possessiveness over his belongings, and anger at other patients coming near him. Mr H was reviewed by a consultant mental health clinician during his admission who determined that his symptoms were related to his dementia rather than psychiatric concerns, so his care was retained in the geriatric care unit.

Over the course of his admission, Mr H and his visiting neighbors progressively informed various nursing and allied health staff about his history of abuse. Staff noted that this abuse appeared to disrupt the development of emotion regulation skills and his sense of belonging with other people. As such, Mr H's neighbors noted that his life was marked by social isolation and behavior designed to reduce the risk of abandonment. Any staff body language that indicated displeasure with him or risk of rejection were highly distressing to Mr H and appeared to trigger an escalation in his behavior.

Using a trauma-informed lens, staff applied this knowledge to tailor care to increase safety and reduce distress for Mr H. Staff used body language cues to constantly demonstrate to Mr H that he was valued and safe in the ward, including maintaining eye contact, using gentle and appropriate touch, and listening and providing validation of emotional distress. These behaviors were helpful for de-escalation when Mr H was very distressed. The staff promoted Mr H's autonomy and sense of control by offering choices wherever possible, particularly when planning for the day. Staff also prioritized transparency with Mr H, telling him exactly where they were going when they left his company and when they would return. Safety was maximized by physically separating Mr H from others in the ward who appeared to be triggering for him. Despite these interventions and their positive effects for Mr H, he maintained significant mental health concerns and was eventually transferred to an Older Person's Mental Health unit with comprehensive discharge information. He remains under the care of a psychiatric team.

### Case 3

Mr D, an 88-year-old Italian man with limited English language, was admitted to hospital after his friend became increasingly worried that he was unsafe living at home by himself. His friend also reported that his “agitated” behavior was making her feel unsafe when he visited her home. After an initial 4 month stay, Mr D was discharged to a residential care facility but then readmitted a month later for a subsequent 5 months. Medical history included mixed dementia, myelofibrosis, alcohol use disorder, and hypertension. During hospitalization, hospital staff had difficulty providing personal care and medical tests (e.g., blood tests) to Mr D as he would become angry when staff encouraged him to wash. Mr D was also particular about which foods were offered, and would become angry at staff if they removed his food tray or touched his non-alcoholic beer without his permission.

While assisting Mr D with shaving one day, he disclosed to a nurse that his sister had made three suicide attempts before dying by suicide by hanging herself in the shower many years earlier. Mr D and another family member found her body. Over time both Mr D and his friend disclosed to staff a range of other challenging experiences throughout his life that had acted as maintaining factors for his heavy alcohol use and difficulty managing his anger. Mr D could become easily overwhelmed with loud noise and activity, and his friend noted that he was a very private person. With permission from Mr D, summary information was shared throughout the team to facilitate development of appropriate care strategies. Interpreters were engaged to better identify Mr D's food and care preferences, and his food and belongings were not touched until he gave permission. Environmental changes were made to promote Mr D's access to privacy and quiet, uninterrupted time. Use of the English word “shower” was identified as a trigger for distress for Mr D and was therefore replaced with “wash” wherever possible. Nursing staff would offer Mr. D a wash with water and a flannel so he could attend to personal care himself where possible. These actions were aligned with trauma-informed care and intended to promote Mr D's sense of autonomy, choice, collaboration, and safety within the ward. The remainder of Mr D's admission to the ward was characterized by a marked reduction in his distress and anger. After several months he was moved to residential aged care and effective care strategies developed in hospital were shared with facility staff during discharge planning to promote transition.

### Case 4

Mr C, a 90-year-old man, was admitted to the ward due to worsening symptoms of Alzheimer's disease which included physical violence to staff and other residents during a respite stay at a residential aged care facility. Mr C did not speak any English and was unable to communicate coherently in his native language. Mr C would become particularly distressed during personal care and showering, when he would do his best to run away from the shower by yelling, shouting, spitting, hitting, kicking and pushing staff. He would not agree to wash himself. Staff would use picture illustrations to help guide during showering but Mr C's distress continued, even with the use of sedative medications as needed.

During a visit to the ward, Mr C's daughter informed the nursing staff that Mr C had been a prisoner of war during the second world war. During this time, he was held down by several men and tortured by having his head forcibly pushed under water. Staff subsequently implemented several strategies to manage Mr C's hygiene without causing distress. Instead of showering, Mr C was offered a hot towel sponge for his daily hygiene. Staff provided reassurance and validation in Mr C's native language and were guided by Mr C regarding how the wash would occur. Staff only showered Mr C when necessary due to fecal incontinence. When showers were necessary, one staff member would communicate with Mr C using simple, repetitive verbal explanations and warm and supportive body language while other staff showered him gently and quietly. Staff avoided wetting Mr C's face. Mr C tolerated these hygiene strategies well and his distress reduced significantly over the course of his admission. The effective strategies were detailed in his discharge plan when he was discharged to a residential aged care facility.

## Discussion

Trauma-informed care is an under-utilized yet potentially beneficial approach to care for older adults and people with dementia in the hospital setting. The cases detailed in this paper demonstrate that the impact of psychological trauma can be wide-reaching in these settings and requires a tailored response that is agreed and delivered by all staff. When effectively implemented, trauma-informed care can promote staff and patient safety, reduce the risk of re-traumatisation, and minimize adverse events.

The relationship between traumatic stress and dementia is complex and bi-directional. Exposure to psychologically traumatic events, particularly when this results in clinically significant post-traumatic stress disorder, is associated with increased risk for dementia (19). At the same time, the symptoms of dementia can trigger the re-emergence or worsening of responses to traumas endured earlier in life (20). The overlap in symptomology of dementia and common trauma responses means that reactions to triggering stimulus may sometimes be mistaken for manifestations of dementia and pathologised as “behavioral and psychological symptoms of dementia” (12). This can result in patients being treated according to clinical guidelines for the management of dementia rather than the response being conceptualized and treated as traumatic stress. For example, trauma-related flashbacks or nightmares may be mistaken for hallucinations and treated with antipsychotic medications. While these medications are commonly prescribed for the management of dementia symptoms, they are associated with unintended side effects and are not recommended for patients experiencing trauma-related responses (12). Patients with comorbid dementia and traumatic stress also risk falling between psychiatric and geriatric care services where these are not integrated (21), as was the case for Mr H.

The cases included here demonstrate how difficult it can be to identify a trauma history and understand its connection to current behavior and distress, particularly with dementia-related deficits in communication, memory, and orientation. Staff came to learn of a trauma history via the patient themselves, their family and friends, through medical records, and at intake assessments. Formal processes to identify trauma-related needs are an essential component of a trauma-informed care setting but are usually insufficient on their own (22). Staff must be attuned to signs of trauma-related distress and able to create trusting relationships that provide a sense of safety for the care recipient (23). Delivery of trauma-informed care must therefore occur at the whole-of-service level, arming staff with the skills and processes to understand trauma and tailor care to meet trauma-related needs (24). The cases presented here demonstrate how professionals without formal mental health training were able to amend their care to acknowledge the role that trauma can play in an individual's experience of receiving health care. This is consistent with the model of trauma-informed care as a system-wide framework in which care providers assume that all care recipients have experienced traumatic stress and are appropriately trained to identify trauma-related needs and amend care to promote safety (25).

Staff applied the principles of trauma-informed care in these cases in a variety of overt and covert ways (Table 1). For example, they promoted the physical and emotional safety of both staff and patients by using methods that were non-triggering for patients (e.g., sponge bath rather than shower), avoiding forceful actions, and tailoring care to meet individual preferences. Staff also avoided using triggering language, and providing patients with a private, quiet, uninterrupted environment. Staff demonstrated their trustworthiness by being transparent in their actions including telling the patient when and how to expect care. Validation was provided using eye contact and appropriate touch. Control and autonomy were prioritized as staff offered choices wherever possible, even where these choices appeared minor. Collaboration was maximized by staff seeking permission from patients before touching their personal belongings, and patient empowerment was prioritized by supporting patients to attend to their own personal care where possible. Staff maintained a respectful and empathic approach even where patient symptoms were very challenging, particularly where the ward culture was characterized by mutual support and debriefing. The provision of interpreters to effectively communicate with patients and respect for cultural customs demonstrated respect for diversity and inclusion.

**Table 1:** Staff actions demonstrated in case studies that promoted the principles of trauma-informed care (23).

**Principle**	**Explanation**	**Staff actions that promoted the principle**
Safety	Ensure physical and emotional safety for staff and clients	• Preferences for care identified and respected • Triggers for distress identified and accommodated (e.g., sponge bath instead of shower) • Use of non-triggering language • Avoiding forceful actions • Providing a private, quiet and uninterrupted environment
Trustworthiness	Maximize trustworthiness through task clarity, consistency and interpersonal boundaries	• Transparency in staff actions • Informing patient where staff member was going and when they would return • Validating emotional distress • Eye contact and appropriate touch
Choice	Maximize client choice and control	• Patients have the option to decline personal care • Offering choices when planning for the day
Collaboration	Maximize collaboration and sharing of power	• Seeking permission before touching patients' belongings • Shared care planning and decision making • Collaborative de-escalation and debriefing activities (e.g., breathing activities)
Empowerment	Prioritize empowerment and skill building	• Supporting patient to attend to own personal care where possible • Self-management • Information sharing to promote consistent care approach
Respect for diversity and inclusion	Acknowledge that trauma and care may be experienced differently according to culture, age, gender, socioeconomic status and religion	• Provision of an interpreter for patient to ascertain needs and preferences • Recognition of impact of gender- and/or race-based violence and respecting care preferences

An essential cross-cutting principle in trauma-informed models of care is information sharing (23). Trauma-informed care settings have processes in place to facilitate appropriate information sharing so that all staff can tailor their care. In the cases reported here, staff used both formal and informal processes to share information including behavior support plans and handovers. Information about triggers and effective care strategies was also incorporated into discharge planning to promote successful return to the community. The ability to share information (including care failures) in a safe and supportive way has been identified by staff in other inpatient settings as a key facilitator of their ability to implement trauma-informed care (22).

The cases reported here were selected as they demonstrate the successful application of trauma-informed care. However, the risk of bias is a commonly reported limitation of the case series methodology (26). It is therefore important to acknowledge that cases are likely to exist where the principles of trauma-informed care may not have been applied effectively or resulted in a less than optimal outcome. Common barriers to the provision of trauma-informed care have included resistance to change among staff, conflicting priorities, low leadership engagement and constraints in staffing, time, and resources (27, 28). An additional limitation of the case series methodology is that of generalisability (26). The approaches illustrated here are not intended to be generalisable to all patients and all healthcare settings but rather provide specific examples that can be tailored or used as a starting point when providing care for older adults who have experienced a psychologically traumatic event. To further explore the benefits of trauma-informed care in this patient group, future work measuring quantitative outcomes would be beneficial such as length of hospital stay, re-admission rates and the administration of sedative or antipsychotic medications.

These cases illustrate that the principles of trauma-informed care can be successfully applied in the care of older adults by staff without formal mental health training. Trauma-informed care is an innovative approach in geriatric medicine, and widespread adoption will require systems-level initiatives including staff training, processes to promote information sharing, environmental change, and improved integration of geriatric and psychiatric care services (22). Much can be learned from mental health services and systems, where trauma-informed care is well characterized and from which toolkits for implementation are available (23). By improving staff skills and organizational systems, older trauma survivors can benefit from tailored, empowering care in which they feel safe and free of distress.

## Data Availability Statement

The original contributions presented in the study are included in the article/supplementary material, further inquiries can be directed to the corresponding author.

## Ethics Statement

Written informed consent was obtained from the individual(s) for the publication of any potentially identifiable images or data included in this article.

## Author Contributions

MC devised the concept of this case series. All authors contributed to the drafting of the manuscript and approved the submitted version.

## Funding

This study was funded by the South Australian Hospital Research Foundation and the Australian Government Medical Research Future Fund. MC was supported by an Early Career Fellowship from the South Australian Hospital Research Foundation (ECF-2019-33-25271) and a National Health and Medical Research Council Medical Research Future Fund Emerging Leadership Investigator Grant (MRFF1194084).

## Conflict of Interest

The authors declare that the research was conducted in the absence of any commercial or financial relationships that could be construed as a potential conflict of interest.

## Publisher's Note

All claims expressed in this article are solely those of the authors and do not necessarily represent those of their affiliated organizations, or those of the publisher, the editors and the reviewers. Any product that may be evaluated in this article, or claim that may be made by its manufacturer, is not guaranteed or endorsed by the publisher.
